# Broadening and Enhancing Bacteriocins Activities by Association with Bioactive Substances

**DOI:** 10.3390/ijerph17217835

**Published:** 2020-10-26

**Authors:** Hassan Zgheib, Djamel Drider, Yanath Belguesmia

**Affiliations:** 1 UMR Transfrontalière BioEcoAgro1158, Univ. Lille, INRAE, Univ. Liège, UPJV, YNCREA, Univ. Artois, Univ. Littoral Côte d’Opale, ICV–Institut Charles Viollette, 59000 Lille, France; hassan.zg@hotmail.com (H.Z.); Yanath.Belguesmia@polytech-lille.fr (Y.B.); 2 Univ. Lille, CNRS, Centrale Lille, Univ. Polytechnique Hauts-de-France. UMR 8520–IEMN, 59000 Lille, France

**Keywords:** bacteriocin, synergism, antibiotics, nanoparticles, phages, essential oils, antiviral, anticancer, antibiofilm

## Abstract

Bacteriocins are antimicrobial peptides some of which are endowed with antiviral, anticancer and antibiofilm properties. These properties could be improved through synergistic interactions of these bacteriocins with other bioactive molecules such as antibiotics, phages, nanoparticles and essential oils. A number of studies are steadily reporting the effects of these combinations as new and potential therapeutic strategies in the future, as they may offer many incentives over existing therapies. In particular, bacteriocins can benefit from combination with nanoparticles which can improve their stability and solubility, and protect them from enzymatic degradation, reduce their interactions with other molecules and improve their bioavailability. Furthermore, the combination of bacteriocins with other antimicrobials is foreseen as a way to reduce the development of antibiotic resistance due to the involvement of several modes of action. Another relevant advantage of these synergistic combinations is that it decreases the concentration of each antimicrobial component, thereby reducing their side effects such as their toxicity. In addition, combination can extend the utility of bacteriocins as antiviral or anticancer agents. Thus, in this review, we report and discuss the synergistic effects of bacteriocin combinations as medicines, and also for other diverse applications including, antiviral, antispoilage, anticancer and antibiofilms.

## 1. Bacteriocins, a Resourceful Antimicrobial Peptides

Bacteriocins are antimicrobial peptides/proteins ribosomally synthesized by numerous bacterial strains during the primary growth phase [1]. They are secreted into the natural environment at a subinhibitory concentration, which is sufficient to inhibit other competitive strains. Of note, the primary function of bacteriocins was originally thought to be their signaling and repelling aptitudes, rather than the inhibition of adverse bacteria. At higher concentrations than natural levels, bacteriocins display strong antimicrobial activity and can induce pore formation and other disturbances of the membrane, or even inhibit the cell division process [2]. Gram-positive and Gram-negative strains producing bacteriocins develop self-immunity systems to protect themselves from the toxicity of their own peptides [3,4]. Moreover, it was shown that DNA coding for bacteriocins, their transports system and immunity genes are generally arranged in operons, which can be located either on plasmids or on chromosomes [5].

Nevertheless, there is no universally accepted scheme of classification of bacteriocins. The first classification, which was proposed by Klaenhammer [1], was established on the basis of structure and modes of action of bacteriocins, and include four classes (Table 1). Thereafter, different classifications were suggested, taking into account further criteria. Thus, Drider et al. [6] considered the biochemical and genetic properties of bacteriocins. Recently Alvarez-Sieiro, et al. [7] considered the biosynthesis mechanism and biological activity of bacteriocins as key elements in their classification (Table 1).

Importantly, bacteriocins are mainly known for their antagonistic properties against both phylogenetically close or distant bacteria [8,9]. However, bacteriocins have been also associated with additional functions such as antiviral, plant protection and anticancer activity [2,10].

Based on their antimicrobial properties, bacteriocins are considered as potential tools to control various infections including postoperative, urogenital, gastrointestinal, respiratory tract and others [2,11].

In addition to their antibacterial potency, few bacteriocins showed ability to inhibit the multiplication of viruses. For example, enterocin CRL35 and subtilosin, showed antiviral activity against some viruses, like herpes simplex virus 1 (HSV-1) or poliovirus (PV-1). These bacteriocins both act by perturbing the late stages of viral replication [12,13]. Furthermore, enterocin B was shown to act against the influenza viruses H1N1 and H3N2 [14]. More recently Małaczewska et al. [15] prospected successfully the antiviral potential of combination of nisin mixed with lysozyme and lactoferrin against the bovine viral diarrhoea virus.

The multifunctionality of bacteriocins includes also their activity as a novel class of anticancer agents [16]. Related to that, LS10 bacteriocin, a defensin-like class IId bacteriocin produced by *Brevibacillus* sp. strain SKDU10, displayed anticancer activity against several cancer cell-lines originated from solid tumors; including H1299 (lung carcinoma), MCF-7 (breast cancer), HeLa (cervical cancer cells) and HT1080 (human fibrosarcoma), at a concentration below 10 μM [17,18].

Furthermore, numerous studies have reported the synergistic effect of bacteriocins when combined with classic antibiotics [19], polysaccharide nanoparticles [20], phages [21], essential oils [22], organic compounds and preservatives [23]. The association of bacteriocins with such molecules was expected to have beneficial effects in various applications including anticancer [24], antibiofilm [25], antimicrobial [26] and antifungal [22]. Of note, a major advantage of bacteriocins-bioactive substances combinations is the low amount of each molecule requested to obtain the desired effect. This amount is expected to be significantly less important than in the case of individual use. These synergistic interactions allow as well to reduce potential toxicity or any adverse effect associated with these molecules [19].

## 2. Antibiotics and Bacteriocins Constitute Promising Synergistic Combinations for Medical Therapy

Antibiotics have been widely used, and at times misused, for decades. Their overprescription has started to limit their effectiveness due to the emergence of antibiotic resistance. Now antibiotics are “dying” and therefore, human and animal health has started to experience untreatable infectious diseases. The combination of antibiotics and bacteriocins is increasingly being pointed out as a key strategy to confront emergent resistant pathogens [19]. This synergistic bacteriocin-antibiotic association has been reported in different circumstances [27,28,29,30,31,32,33,34,35,36]. The combination of these molecules offers key incentives as the total amount of antibiotics administered is reduced as is their potential cytotoxicity [27,32]. Overall, the data gathered from some studies indicated the effectiveness of bacteriocins-antibiotics combinations to control resistant clinical pathogens such as methicillin-resistant *Staphylococcus aureus* (MRSA), *Enterococcus faecium* and *Clostridium difficile* [30]. Of note, the latter organism is responsible for *C. difficile*-associated diarrhoea (CDAD), which is attributed to the perturbation of the gut microbiota resulting from the overuse of antibiotics [37]. Interestingly, Le Lay et al. [38] established the inhibitory activity of nisin on *C. difficile* by targeting the vegetative cells and the spore germination process. On the other hand, Hanchi et al. [30] established the synergistic effect of combination of reuterin and durancin 61A on the growth of *C. difficile.* Further bacteriocins, like thuricin CD [39], lacticin 3147 [40] or actagardine A [41], are active as well against *C. difficile*, which opens a window for treatment of infections associated with this pathogens using bacteriocins.

Furthermore, Danesh et al. [42] showed that haloduracin, a lantibiotic bacteriocin, associated with chloramphenicol displayed a wide spectrum of activity against pathogens such as *Enterococcus faecalis*, *E. faecium*, *S. aureus* and even against different strains of *Streptococcus* [42]. Recently, the combination of a bacteriocin, produced by *Lactobacillus acidophilus*, with ceftazidime, imipenem and minocycline antibiotics decreased the required minimal inhibitory concentrations (MICs) values of these antibiotics against a strain of *Stenotrophomonas maltophila* S19, which was previously shown to be resistant to these three aforementioned molecules [43]. Similarly, Al Atya et al. [27] demonstrated the synergetic activities of two class IIb bacteriocins, enterocins DD28 and DD93, associated with kanamycin and erythromycin against a clinical strain of MRSA. In direct line, the combinations of another bacteriocin, the lacticin 3147, with penicillin G or vancomycin and nisin z with methicillin, successfully inhibited the growth of MRSA strains [44]. Another synergistic effect of combining nisin A and cefazolin was reported against mastitis pathogens and also *S. aureus, E. faecalis, Staphylococcus intermedius*, *Streptococcus agalactiae*, *Streptococcus dysgalactiae* and *Escherichia coli* [45].

Currently, urinary tract infections (UTIs) are seen as a global public health issue caused by *E. coli* strains and other bacteria including *E. faecalis*, *Staphylococcus saprophyticus*, *Klebsiella*, *Citrobacter*, *Pseudomonas* and *Proteus mirabilis* [46,47]. The high rates of occurrence of UTIs due to these pathogens and their increasing antibacterial resistance conducted to unfavorable outcomes, like complicated UTIs (cUTIs), pyelonephritis and severe urosepsis [48,49]. Thus, UTIs are infections needing the development of new therapeutic agents. The combination of garvicin KS and nisin with tetracycline or polymyxin B has revealed effective synergies against UTIs provoked by multidrug resistant Gram-positive and Gram-negative bacteria [50,51]. Additionally, a recent study conducted by Biswas et al. [52] showed that bacteriocin-antibiotic combinations have synergistic effects against β-lactamase-producing clinical pathogens.

Bacteria living in biofilms structures are difficult to treat [53]. These aggregates of organized bacterial cells in complex structures provide efficient protection from antibiotics and detergents action, and therefore limit the efficacy of these antimicrobial agents [54]. The use of bacteriocins in association with classic antibiotics has shown great efficiency in terms of potent inhibition of biofilm formation and restoring antibiotic-sensitivity to the bacteria endowed in these elaborate biological structures [27]. Indeed, the combination of nisin with polymyxins reduced dramatically the required concentrations of polymyxins to treat *Pseudomonas aeruginosa*, which is known for its ability to form biofilms in the lungs of patients in intensive care [29,55]. This significant reduction in the concentrations was shown to be related to the biofilm-penetrating abilities of nisin, making it a good agent for eradicating or preventing biofilm communities on medical devices and hospital equipment [56]. Furthermore, the recent study conducted by Angelopoulou et al. [57] showed a synergistic effect between nisin A and vancomycin against biofilms of multidrug resistant *S. aureus* isolates from human milk.

The association of bacteriocins and antibiotics has provided a new life to conventional antibiotics which had started to be ineffective against multidrug resistant bacteria. These combinations have extended their spectrum of activity and thus opened a new window for the treatment of infectious diseases.

Beyond their classical antimicrobial activities, bacteriocin-antibiotic associations displayed novel unexpected and very interesting activities like the antitumor one, which could be considered as new anticancer therapy options [16]. Despite the increasing successes in some areas, the traditional treatments for most cancers are facing to increasing limitations. In spite of the availability of various methods for cancer treatments nowadays, which includes chemotherapy, surgery and radiotherapy, the rate of mortality remains consistent with significant side effects [58]. Globally, malignancy resulting from abnormal cells divisions and uncontrolled cell proliferation is one of the most difficult disease areas to treat. In addition to the nonspecificity of chemotherapy, the resistance of cancer cells towards this treatment is growing; therefore, a novel approach to anticancer therapy is urgently needed [16]. Recent studies demonstrated the antitumour activity of some bacteriocins against diverse cancer cell lines [18]. In direct line, nisin and its derivatives peptides exhibited in vitro and in vivo antitumor potential on mice model in head and neck squamous cell carcinoma (HNSCC) [59,60]. Interestingly, during another study the combination of nisin with doxorubicin, an anthracycline antibiotic, has effectively decreased the mean skin tumor volume and tumor burden in mice during in vivo assays. These observations advocate for the possible use of the nisin/doxorubicin combination to help developing alternative strategies to combat drug resistance in skin cancer cells [61].

## 3. Nanoparticles for Carrying and Improving the Bacteriocin Activity

**Figure 1:**
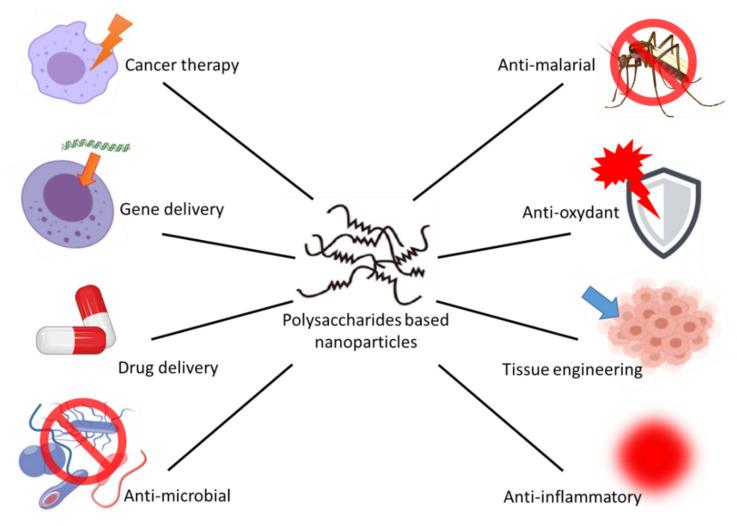
Schematic representation of the nano-biotechnological applications of polysaccharide-based nanoparticles (NPs).

Nanoparticles (NPs) are ultrafine particles with size range from 1 to 100 nanometers in diameter [62]. The surface area of the NPs can determine how they interact with their surroundings (solid, liquid or gas). As the size of the particle decreases, its surface area per unit volume increases. Due to the nanometric scale of these particles, they possess unique physical and chemical properties compared to those of solid materials at the micro- and macro-scales [63]. The increasing interest for NPs has conducted for the design of nanoparticles from different materials with different shapes [64]. NPs are distinctly different from micro- and macro-scale particles. They are influenced by physicochemical properties such as size, shape, charge among others [65]. NPs can be divided into two families, which are nondegradable NPs (quantum dots, fullerenes and metallic NPs) displaying cytotoxic effects [66,67] and degradable NPs such as polysaccharide ones, which are generally safe [68,69]. Additionally, these functional polymers have a wide range of uses, including pharmacological and biological ones (Figure 1 and Table 2) [70,71,72,73].

NPs with drug delivery properties are of major interest [71,74]. NPs can entrap drugs inside their structures or adsorb them onto their surfaces. Moreover, NPs can improve the low stability of proteins and nucleic acids in biological environments and overcome their limited passage through the biological barriers and thereby enhance their delivery to the target site [71]. Of note, the temperature, pH sensitivity and biodegradability of these particles can confer a controlled release profile. In addition NPs can also reduce adverse side effects of drugs and enhance their effectiveness [71].

Due to these interesting properties, NP’s have been extensively studied for pharmaceutical, biological and medical uses. Research on this topic is currently focused on the use of NPs as protectors for the delivery of sensitive elements to the targeted sites without being degraded by pH, enzyme degradation and oxidation [75]. Several bioactive components including amino acids, fatty acids, proteins…etc. have been reported for their potential properties for inflammations, coronary heart disease (CAD) and regulation of blood pressure [76]. Nevertheless, their poor stability in the gastrointestinal tract, low solubility and bioavailability may limit their efficacy. NPs may balance these weaknesses by enhancing these molecules stability and dispersibility, protecting them thereof from digestion and increasing their absorption into the bloodstream [76]. Thus, the combination between nanotechnology and biotechnology can lead to novel strategies, which can easily overcome these drawbacks especially in the biological systems [77]. Combinations of bacteriocins and NPs designed as nano-antibiotics formulations offer many incentives. Indeed, the use of bacteriocins can be limited by their sensitivity to proteolytic enzymes (pancreatin, trypsin, etc.). This can be resolved by adsorbing bacteriocins on NPs [78]. Furthermore, recent studies pointed out the antibacterial [79], antibiofilm [80] and anticancer [81] properties of nano-antibiotics (Table 3).

## 4. Combined Effects of Bacteriocins and Bacteriophages

Bacteriophages (phages) can undergo two different life cycles of reproduction: a lytic cycle or a lysogenic cycle [83]. Unlike lysogenic ones, lytic phages have a strong bactericidal effect. Once they infect their target they keep replicating until the targeted bacterial population is eliminated, which means that a low phage count, in one administration, is sufficient to eliminate the pathogens [83]. Bacteriocin-bacteriophage combinations have recently been investigated for various applications [21,26,84,85]. Recent studies highlighted the synergistic interactions of coagulin C23 (a class II bacteriocin) with two bacteriophages named FWLLm1 and FWLLm3, against the foodborne pathogen *L. monocytogenes*. The combination of FWLLm1 phage and coagulin C23 has significantly reduced the levels of *L. monocytogenes* 2000/47 (to less than 10 CFU/mL) after 96 h of food matrix storage at 4 °C [21]. However, the combination of FWLLm3 phage with the same bacteriocin failed to inhibit the growth of *L. monocytogenes*, which was attributed to a resistant mutant to the phage. Of note, the authors reported that the development of resistance was much lower when the antimicrobials were combined, explaining the synergistic effects observed in this study [21]. In direct line, Baños et al. [84], determined the ability of bacteriophages P100 and enterocin AS-48, a cyclic bacteriocin to control the growth of *L. monocytogenes* present in two raw fish flesh (salmon and hake). Thus, AS-48 alone reduced the growth of *L. monocytogenes* cells counts, compared to the untreated sample, by 1.9, 2.55, 2.8, and 2.8 log CFU/cm^2^ (in hake) and by 1.68, 2.79, 2.9, and 3.13 CFU/cm^2^ (in salmon) at one, two, three, and seven days, respectively. Despite the low reductions attributed to phage P100, conversely to bacteriocin AS-48, their combination (AS-48/P100) permitted the eradication of the pathogens from raw salmon and hake flesh after one to two days of treatment. In another study, combination of bacteriophage treatment with bacteriocins was successfully tested against *C. perfringens* strains, isolated from chicken and swine feces [26], and against *S. aureus* KCTC 3881 reference strain [85].

## 5. Bacteriocins-Essential Oils Synergy are Active Against Pathogenic Bacteria

The antibacterial activity of essential oils (EOs) and their promising properties as antimicrobial agents has been demonstrated in many studies, and their applications are anticipated to be meaningful for the food industry and medicine [86]. EOs can be used in combination with other antimicrobial agents such as bacteriocins to enhance their effects, and reduce resistance as well as doses required for such activity [87,88,89]. Mehdizadeh et al. [88] reported the antimicrobial effect of three EOs, extracted from basil (*Ocimum basilicum*), sage (*Salvia officinalis*) and ajowan (*Trachysper mumammi),* in combination with nisin against *E. coli* O 157 and *S. aureus*. Data obtained exhibited synergistic interactions for all these combinations against *E. coli*, and the highest one was observed for nisin and *Salvia officinalis* EO combination. Notably, no change was observed in the antimicrobial activity of these combinations towards *S. aureus* [88]. Further, Ay and Tuncer [87], showed that when nisin is combined with carvacrol, a monoterpenoid phenol issued from oregano “*Origanum vulgare*” EO, and ethylene diamine tetra-acetic acid (EDTA), a chelating agent used in the food industry, the counts of *Salmonella* Typhimurium were reduced to undetectable levels [87]. Moreover, nisin, when combined with cinnamaldehyde (CA), a phenylpropanoïd isolated from cinnamon (*Cinnamomum verum*), displayed synergistic antimicrobial activity against thirteen foodborne isolates of *S. aureus* [89]. LIVE/DEAD Bac Light and scanning electron microscope (SEM) assays performed on treated bacteria cells revealed greater damage, resulting from the combination of the bacteriocin and the EO, on both cell wall and cell membrane compared with treatments by nisin or CA lonely [89].

As indicated before, biofilms allow bacteria, including pathogens, to be protected from a number of antimicrobials. Indeed, when biofilms have reached the irreversible attachment stage, it is extremely hard to penetrate or remove them [90]. Nonetheless, investigations requesting bacteriocins-EOs as antibiofilms agents have been developed. Related to that, Smith et al. [91] revealed the synergism between M21A; a nisin bioengineered peptide (0.1 μg/mL), and CA (35 μg/mL) on biofilms of *L. monocytogenes* [91]. In addition, Iseppi et al. [25] revealed the effectiveness of thyme (*Thymus vulgaris*) and sage (*Salvia officinalis*) EOs, alone or in combination with the bacteriocin bacLP17 against 12 strains of *L. monocytogenes*. The authors reported that the best antibiofilm effect was observed with the combination of EOs and bacteriocin, compared to both individual treatments and controls [25].

Of note, Issouffou et al. [22] showed the potent activity of the combination of EOs and bacteriocin against 18 spoilage microorganisms (nine strains of yeasts and nine strains of bacteria). The nine spoilage bacteria were *Serratia marcescens, Klebsiella variicola, E. faecalis, Lactococcus lactis* subsp. *lactis* and *Klebsiella pneumoniae*, whereas the spoilage yeasts were *Hanseniaspora opuntiae*, *Pichia* aff. *fermentans*, *Candida metapsilosis*, *Pichia kundriavzevii* and *Kodamaea ohmeri* (Table 4). The results indicate that the combination of cinnamon and enterocin KT2W2G displayed a synergistic and broad action against the selected spoilage microorganisms. Notably, the use of enterocin KT2W2G alone did not display any activity [22]. The use of EOs in food matrixes needs to be further evaluated as EOs could modify the organoleptic properties if used at concentrations exceeding a certain threshold [92]. Nevertheless, combination of bacteriocins with EOs allows the diminishing of the required concentrations of both compounds and therefore might circumvent this issue. In conclusion the combination of bacteriocins and EOs stands as a promising and potent protective strategy that deserves to be deeply investigated.

## 6. Conclusions

Bacteriocins have been steadily reported as alternatives to traditional antibiotics. In this review, we highlighted their multifunctionality and their synergistic effects once they were used in combination with other molecules and antimicrobial agents. Such combinations can enhance the potency of bacteriocins and lead to novel formulations such as nano-antibiotics that can be used in the near future to fight infections, which are defying the aging and fading of traditional antibiotics. Combination therapies enhances the properties of bacteriocins which become more stable, with better solubility, bioavailability and efficiency. Thus expanding their spectrum of use to antiviral, antibiofilm and anticancer fields. Finally, the extent area of the multifunction of bacteriocins, along their exciting synergistic effects, could be helpful to extend the research field of their associations with other bioactive substances, once the safety and efficiency of such combinations are assessed in vitro and in vivo.

## Figures and Tables

**Table 1:** The main classification schemes of bacteriocins.

Bacteriocins Classification Scheme According to:		
Klaenhammer [1]	Drider et al. [6]	Alvarez-Sieiro et al. [7]
**Class I** Small modified peptides < 5 kDa; containing specific amino acids (lanthionine and dehydrated residues.	**Class I** Small peptides with post-translational modification (lanthionines and dehydrated residues) **Remark:** This class was subdivided in two types: **Type A** (elongated molecules with molecular mass < 4 kDa) **Type B** (globular molecules with molecular mass between 1.8 and 2.1 kDa)	**Class I** Ribosomally produced and post-translationally modified peptides (RiPPs) (<10 kDa) **Remark:** Five subclasses were assigned to class I bacteriocins: **Ia:** lanthipeptides (types I, II, III, and IV) **Ib:** Cyclized peptides (Enterocin AS-48) **Ic:** Sactibiotics **Id:** linear azol(in)e-containing peptides **Ie:** Glycocins **If:** Lasso peptides
**Class II** Small unmodified peptides (<10 kDa); thermostables; acting by pore formation in the membranes	**Class II** Small heat-stable unmodified peptides (<10 kDa) **Remark:** Three subclasses were retained **IIa:** Pediocin-like bacteriocins **IIb:** Two-peptide bacteriocins **IIc:** Other peptide bacteriocins including cyclized ones (enterocin AS-48)	**Class II** Unmodified peptides (<10 kDa) **Remark:** Four subclasses were proposed **IIa:** Pediocin-like bacteriocins **IIb:** Two-peptides bacteriocins **IIc:** Leaderless bacteriocins IId: Non pediocin-like single-peptide bacteriocins (ex: laterosporulin) including cyclized ones (enterocin AS–48)
**Class III** Proteins with molecular weight > 30 kDa; enzymatic activity	**Class III** Large, heat-labile (>10 kDa) protein	**Class III** Thermo-labile (>10 kDa) bacteriolysins and non-lytic bacteriocins
**Class IV** Proteins attached to sugars or lipids		

**Table 2:** Polysaccharides and the applications of their NPs in delivery systems .

Polysaccharides	Origin	Applications of Its NPs
**Alginate**	Extracted from brown algae (*Phaeophyceae*)	Gene and drug delivery system, tissue engineering, etc.
**Cyclodextrin**	Enzymatic conversion of starch	Gene and drug delivery, controlled drug release
**Starch**	Higher plants	Insulin controlled release & drug delivery
**Pectin**	Extracted from the middle lamellae of plant cells	Wound healing, delivery of amino acids and drugs
**Chitosan**	Extracted from the cell walls of fungi and the exoskeleton of crustaceans (crabs, shrimp)	Nanocarrier in drug delivery and antimicrobial
**Cellulose**	Cell wall of green plants	Nanodelivery for oral protein, for anticancer drugs, etc.

**Table 3:** Synergistic effects of combining nisin and NPs against various applications.

Bacteriocin-NPs Combinations	Applications	Targets	References
**Nisin-alginate-chitosan NPs**	Antibacterial	*L. monocytogenes*	[79]
**Nisin-alginate-chitosan NPs**	Antibacterial	*S. aureus* *L. monocytogenes E. coli* *S. Typhimurium*	[82]
**Nisin-nano vesicles**	Antibiofilm	*S. aureus* *L. monocytogenes* *E. coli, P. aeruginosa*	[80]
**Nisin-PLA-PEG-PLA NPs**	Anticancer	Cancer cell lines: Gastrointestinal (AGS and KYSE-30) Hepatic (HepG2) Blood (K562)	[81]

**Table 4:** Antimicrobial activities of bacteriocin-essential oil (EO) combinations.

Bacteriocin-EO Combinations	Target Bacteria/Yeast
Nisin-Salvia officinalis	*E. coli* O 157
Nisin-Carvacrol	*S. Typhimurium*
Nisin-Cinnamaldehyde	*S. aureus*
M21A- Cinnamaldehyde	*L. monocytogenes*
Bacteriocin bacLP17- *Thymus vulgaris*, *Salvia officinalis*	*L. monocytogenes*
Enterocin KT2W2G- Cinnamon	**Bacteria:***Serratia marcescens, Klebsiella variicola, Enterococcus faecalis, Lactococcus lactis* subsp. *lactis*, *Klebsiella pneumoniae*. **Yeasts:** *Hanseniaspora opuntiae*, *Pichia* aff. *fermentans*, *Candida metapsilosis*, *Pichia kundriavzevii* and *Kodamaea ohmeri*.
